# Prevalence of Gastrointestinal Parasites in Local and Exotic Breeds of Chickens in Pankrono–Kumasi, Ghana

**DOI:** 10.1155/2019/5746515

**Published:** 2019-09-02

**Authors:** Philip Asumang, Justice Akoto Delali, Francis Wiafe, Zeba Kamil, Gadafi Iddrisu Balali, Vera Afua Dela Gobe, Wilson Nketiah Siaw, Grace Pinamang

**Affiliations:** ^1^Department of Science Education, University of Education, Winneba, Ghana; ^2^Department of Theoretical and Applied Biology, Kwame Nkrumah University of Science and Technology, Kumasi, Ghana; ^3^Department of Science, St Monica's College of Education, Ghana

## Abstract

The world's poultry population is on the ascendency as a result of the high demand for poultry product by consumers. In Africa, poultry meat is estimated to represent almost 25% of all meat, whereas in some areas it covers 100% of the animal protein available. The high demand for poultry products has led to an increase in poultry production in almost all African countries including Ghana, with the domestic chicken being the most kept. The sector has been reported to have recorded a drop in production, partly due to infection of birds by diseases, causing organisms including parasites. The study conducted was to investigate the prevalence of gastrointestinal parasites in local and exotic breeds of chickens in Pankrono–Kumasi in the Ashanti Region of Ghana. Two hundred (200) cloacae of slaughtered birds were collected from slaughtering units in the study area and the faecal samples were examined for the eggs/cysts of gastrointestinal parasites using the simple flotation technique and microscopy. Nematodes and cestodes were recovered in 131 (65.5%) of the samples examined with* Ascaridia galli* recorded as the most prevalent. Some of the nematodes include* Ascaridia galli *65 (32.5%),* Heterakis gallinarum *38 (19.0%), and* Capillaria *spp. 29 (14.5%). Some cestodes were* Raillietina *spp. 19(9.5%)* and Choanotaenia infundibulum *5 (2.5%) with* Prosthogonimus *spp. 3 (1.5%) being the only trematode recovered. The local breeds recorded a percentage prevalence of 76.0%, making them the most susceptible breed to gastrointestinal parasites. The results obtained attest to the reason behind the reduction in poultry production. It is therefore recommended that farmers are educated on farm managerial practices that will reduce the risk of infection and help increase production to meet the demand of consumers.

## 1. Introduction

The total population of poultry in the world has been estimated by the Food and Agricultural Organization of the UN [1] to be 14.718 million with 1.125M distributed throughout Africa, 1.520M in South America, 6.752M in Asia, 9 M in Oceania, 3.384M in North America, and 1.844M in Europe [2]. The most commonly kept poultry are the domestic chicken (*Gallus gallus domesticus*) [3, 4]. Based on the number of animals, poultry represent the largest domestic animal stock in the world [5]. This has been demonstrated by the number and fact that during the last three decades, egg production has doubled and poultry meat production has tripled whereas there is no much increment in livestock production due to higher demand for poultry products [6]. In Africa, poultry meat is estimated to represent almost 25% of all meat, whereas in some areas it covers 100% of the animal protein available [7].

Ghana's overall livestock production has been on the rise since 2000 largely as a result of the exponential growth of the poultry sector in the Southern region [1]. While the total cattle production increased by 8 percent between 2000 and 2007, poultry production increased by more than 80 percent for the same period [7], resulting from the establishment of the integrated poultry project in Accra by the Government of Ghana in the 1960s and the high demand of poultry meat by the Ghanaian populace [8].

In Ghana, the village or backyard poultry production system is the most prevalent one, complemented by the commercial production system. The country's village poultry population was estimated at 12 million [9] and in 2005 at over 20 million (FAOSTAT), accounting for 60 – 80% of the national poultry population being kept all over the country in the rural and periurban areas [10], with the Upper East, Upper West, and the Northern regions being the concentrated regions compared to exotic breeds.

Research work by [11] avers that commercial poultry production has made substantial progress during the last sixty years in both Western and Central Africa. However, the development of vibrant poultry sectors in these countries is dependent on costly imported day-old chicks from high-performance hybrid stocks, balanced feeds, drugs, and vaccines. For example, the 11 hatcheries in Ghana are operating at only 38% of their total production capacity [12]. The commercial production system mostly consists of the exotic breeds which are kept for commercial purposes and are more abundant in the urban areas of the Greater Accra, Brong-Ahafo, and Ashanti regions where the market for their product exists and the climatic conditions are favorable [13]

These birds provide man with high nutritional values through the consumption of their meat and/or eggs and other socioeconomic benefits which cannot be overemphasized [13, 14]. However, the supply of poultry products lags behind demand. With a projected national poultry population of 33,525,369 according to [15], it is estimated that a total of 36, 184 mt of poultry meat was produced in 2005. According to [16], these data represented between 18% and 24% only of the total meat demand of Ghanaians. This led to Ghana's national per capita animal protein consumption being one of the lowest in Sub-Saharan Africa, estimated at some 53 g per day [17] which is lower than the recommended 65 g. Poultry meat and eggs together account for only 0.60 percent of the daily calories consumed [18]. It has been estimated that, consumption of poultry products in Ghana consist of 1.2kg of meat and 12 eggs per person in a year as compared to the world's average of 9.7kg of meat and 154 eggs per person per year [19]. This is partly attributed to the losses encountered in the poultry industry which have been linked to outbreak of disease causing agents such as viruses, bacteria [20], and mostly parasites [21] as it has been estimated that more than 750 million chickens, guinea fowls, and ducklings in Africa die each year as a result of various infections [22].

Poultry production in the Ashanti region accounted for 28.07 percent of the total poultry production in Ghana in 2009 which was second to the Brong-Ahafo region 29.62 percent [23]. This indicates the rise in the consumption of poultry product in the region especially the capital city where most farmers bring their product for market. The region has however recorded a decline in production due to infections of birds by disease causing agents with parasites being the most prominent ones [24, 25]. Although some reduction in bird's parasitic infection has been achieved in commercial production system due to improved housing and hygienic and management practices, the prevalence of gastrointestinal parasites is still very rampant [26, 27].

The range of feeds fed on by the domestic chicken in the traditional production system, from grains, fruits, to insects may harbor infective stage of parasites thereby predisposing them to parasitic infections, particularly gastrointestinal parasites [28, 29]. The famer is equally at risk of cross-infection as he carries out his managerial practices on the farm [30].

Good knowledge of the parasites of domestic chickens, species composition, and predilection site is essential for prompt disease diagnosis and treatment [31]. This study, therefore, explored the gastrointestinal parasites of the domestic chicken (*Gallus gallus domesticus*), both local and exotic in Kumasi-Pankrono in the Ashanti Region which to the best of our knowledge is being conducted in the area for the first time. The study seeks to specifically find out (1) the prevalence and types of parasites in the gastrointestinal tract of chickens, (2) the breed that is more prevalent to gastrointestinal parasites, (3) the sex of chickens that is more prevalent to gastrointestinal parasites.

The outcome of the study will serve as a complement to the already existing studies on the subject matter and other related fields. Poultry farmers will be enlightened on the prevalence of gastrointestinal parasites that both local and exotic birds are susceptible to so that measures can be implemented to improve commercial and free-range production systems of poultry. Finally the public will also be informed on the susceptibility of chickens to parasite infections and the risk involved in consuming undercooked meat from poultry.

## 2. Materials and Methods

The study was conducted in Pankrono, a suburb of Kumasi in the Ashanti Region. It has geographical coordinates of 6^0^ 45′ 0” North and 1^0^ 36′ 0” West. It has a population of 60,917 and is approximately 9.8km away from the Kumasi Central Business District. Most of its inhabitants are civil servants, whiles some are petty traders and a few are being farmers.

The study was conducted between January and March 2017. For the purposes of the study, feacal remains in the lower ends of the large intestine of slaughtered chickens were considered.

Two hundred (200) cloacae of slaughtered chickens comprising of 100 locals (50 males and 50 females) and 100 exotic (50 males and 50 females) breeds were collected at random in labelled sample containers and transported to the University of Education, Mampong Campus for laboratory examination.

In the laboratory, the cloacae were cut open and the faecal samples were scrapped into a universal bottle and analyzed for eggs/cyst of intestinal parasites qualitatively using supersaturated saline flotation technique [28, 32].

The floatation medium was prepared by dissolving 400 g of NaCl in 1000 ml of warm distilled water. The procedure was conducted by adding 10mls of the floatation medium to the feacal sample in the universal bottle and stirred with a rod. The mixture was then filtered through double layered gauze into a test tube and more media was added until a meniscus was formed. A coverslip was placed gently on the test tube and allowed to stand on a level surface for at least 10 – 20 minutes. The coverslip was carefully removed and placed on a glass slide and examined immediately for parasite eggs under x10 and x 40 objective lens. Identification of the eggs was aided by the addition of Lugol's Iodine solution to the sample on the glass slide.

The data obtained from the laboratory examination of the samples were collected using Ms Excel 2015. The Chi-square test (X^2^) was used for comparison of prevalence and mean intensity among groups for statistical similarities or differences at a significance level of P < 0.05 and 95% confidence interval.

## 3. Results

Out of the 200 samples examined, 131 (65.5%) were infected with various species of gastro-intestinal parasites (Figure 3), comprising of 5 species of nematode and 4 species of cestode and a species of trematodes. The nematode parasites recorded were* Ascaridia galli *65(32.5%),* Heterakis gallinarum *38 (19.0%),* Capillaria *spp. 29 (14.5%)*, Tetrameres americana *5(2.5%), and* Gongylonema ingluvocola *4(2.0%). The cestodes recovered were* Raillietina *spp. 19(9.5%)*, Choanotaenia infundibulum *5(2.5%)*, Davainea proglottina *4(2.0%),* and Prosthogonimus *spp. 3(1.5%) as the only trematode (Table 1).* Davainea proglottina* was discovered in only female birds of both breeds whereas all the other parasites were seen in both sexes (Figure 2). Nematodes were the most prevalent of the three groups present in the samples examined. However, none of the parasites recovered were known to be zoonotic.

Among infected birds (131),* A*.* galli*,* H. gallinarum* and the* Capillaria *spp. were the most prevalent with a percentage prevalence of 49.63%, 29.0%, and 22.12%, respectively (Table 1). The most prevalent among nematodes was* A*.* galli* (46.1%) and in cestodes,* Raillietina* spp. was the most prevalent (65.0%).

**Figure 1:**
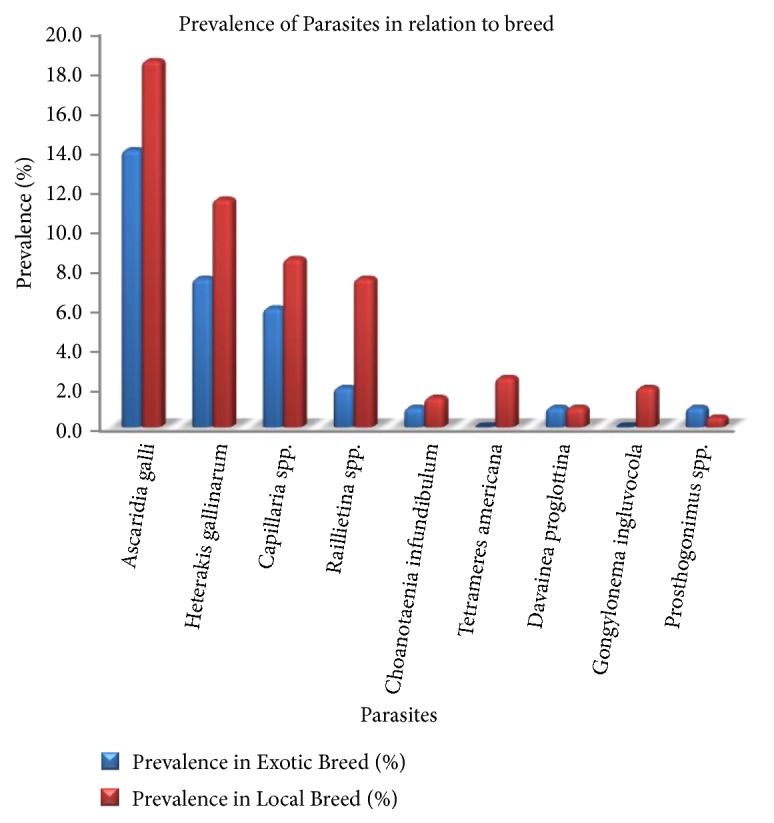
A chat showing prevalence of gastrointestinal helminths of local and exotic breeds of chicken in Pankrono-Kumasi Ghana.

**Figure 2:**
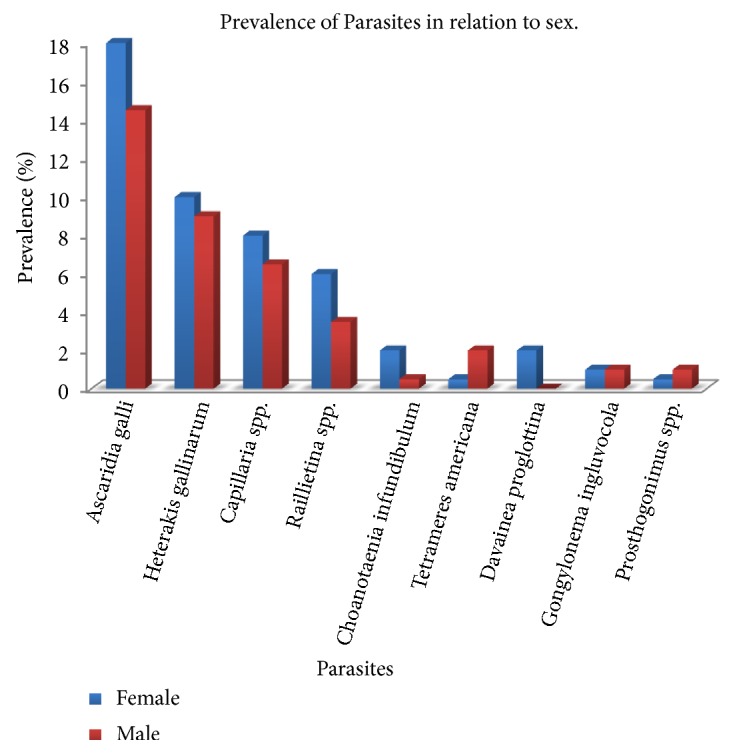
A chat showing the prevalence of gastrointestinal helminth in relation to sex.

**Figure 3:**
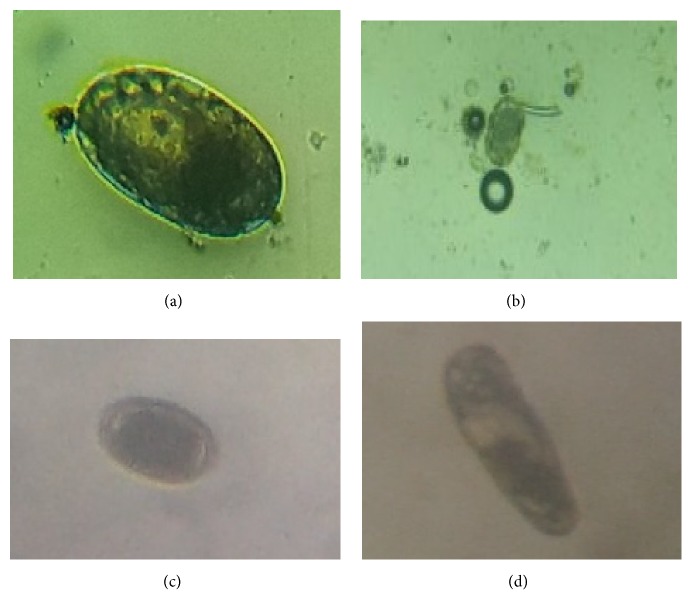
Photographs of some eggs of gastrointestinal helminths of local and exotic breeds of chicken in Pankrono-Kumasi Ghana. (a)* Capillaria  *egg, (b)* H. gallinarum*, (c)* Ascarid egg*, and (d)* Choanotaenia infundibulum*.

The overall prevalence of infection in local breeds (76.0%) was significantly higher than the exotic breeds (55.0%). This is not uncommon because of their free range mode of managerial practices which allows them free access to virtually all types of environment and hence, predisposing them to various forms of infections [33]. The three most prevalent species have yet again showed their dominance in both breeds with* A*.* galli* recording a percentage prevalence of 14.0% in exotic breeds and 18.5% in the local breeds,* H. gallinarum*, 7.5% and 11.5%, and* Capillaria *spp. with 6.0% and 8.5% in the exotic and local breeds, respectively (Figure 1).

Even though there is no significant difference in the overall prevalence of the parasites between females (48.0%) and males (38.0%), the parasites showed some degree of preference for female birds as higher infection rate was observed in females than males in both breeds. The prevalence in the females of the local breed (30.0%) was however higher than observed in the females of the exotic breeds (18.0%).

## 4. Discussion

The overall prevalence of infection with gastrointestinal parasites recorded in this study was 65.5%. This is in relation to the 63.6% reported by [26] in Makurdi and slightly above 59.64% by [34] in Ethiopia. The recorded prevalence is, however, lower than the 92% by [35] and 81.5% reported by [14] in Giwa, Kaduna State of Nigeria. This could be related to the differences in the management system, control practice in farms, and seasonal differences in the study area [3].

The study revealed nematodes and cestodes as the most common intestinal parasites of chickens. This is in accordance with the works of [36] in Zaria, and [14, 36] in which cestodes and nematodes were implicated as the major cause of helminth infection in domestic chickens. Cestodes generally undergo an indirect mode of transmission where they make use of intermediate host such as ants, grasshoppers, and beetles to perpetuate their transmission. These organisms serve as food for scavenging birds and hence transmit the infective stage of the parasites to the bird upon ingestion. The high prevalence of nematodes and cestodes recorded in the local and exotic breeds gives an indication that neither breed is spared by the raid of gastrointestinal parasites in the study area. Their prevalence also indicates the availability of their infective stages in the study area and the ability of the infective stages to withstand environmental conditions for a long time before they are taken in by the host.

Although* A. galli*,* H. gallinarum*,* and Capillaria *spp. infested both local and exotic breeds in this study,* A. galli* had the higher prevalence and this is in consonance with several studies which indicate the species as the commonest and most important helminth infection of poultry [28, 37]. This is not surprising as* A. galli *have a direct mode of transmission and their eggs are very resistant to the environment and can survive on the outside for a long time. The eggs are passed out in the faeces of the host and develop into the infective stage in the open, contaminating feed and water sources. New hosts become infected when they ingest the infective eggs from these sources. In the deep litter system, the eggs can probably remain infective for years depending on the temperature, humidity, pH, and ammonium concentration and so where proper managerial practices are not in place, feed and water sources of birds can easily be contaminated, as farm handlers can transport the eggs of these parasites from other sources to the farm site.

The recorded prevalence (76.0%) in the local breeds over the exotic (55.0%) in the study agrees with previous reports by [21, 37, 38] which conforms to the phenomenon that local breeds are more predisposed to infections due to their roaming and feeding habits where the chickens scavenge during the daytime, with free access to the ground, air, fruits, leaves, roots etc. and therefore have greater contact with faeces and intermediate host organisms such as earthworms, slugs, and snails, exposing them to a wide range of infections. Unlike the local breeds, the exotic breeds are mostly in a confinement and so have limited access to these intermediate hosts and are also prone to regular deworming exercises which minimize the risk of infection. The duration for the local breed to reach table size is much longer compared to the exotic breeds which are fed usually on artificial diets. This could also be a likely reason for the higher infection in the local breeds which continue to accumulate parasites in the system as well as the poor management practices inherent in free range system [33]. It could be said that the exotic breeds were in confinement where good managerial practices such as regular deworming might have occurred and hence recording a low prevalence. This indicates that in areas where the keeping of local breed is more common, parasite infestation will be a threat to the socioeconomic survival of many farmers because there will be a high cost of production with low output [21].

Although the exotic breeds recorded the least prevalence rate in the study area, the figure obtained (55.0%) is high for a commercial production system. This presupposes therefore that the handling and/or management practices at these production sites are woefully poor. The unhygienic conditions in these sites have created a conducive environment for the survival of the larvae of these parasites.

The study revealed that female birds recorded many gastrointestinal helminths than the males in both breeds even though there is no significant difference in the figures obtained. This could be by chance or may be related to their feeding habit as the females are known to be more voracious in their feeding habits especially during egg production than the males which remain largely selective [25]. Though some zoonotic parasites can be found in chickens, the ones recovered in this study were not zoonotic and therefore might not pose any risk of infection to handlers.

## 5. Conclusions

The overall prevalence of gastrointestinal helminths was 65.5% with* Ascaridia galli* found to be the most prevalent. The helminths discovered were mostly nematodes, cestodes, and a trematode. Local breeds were found to have many gastrointestinal helminths than the exotic breeds as they recorded the most prevalence. In relation to sex, the results revealed that female birds were more prone to the parasites than the males as observed in both breeds.

## 6. Recommendations

It is recommended that farmers under the free-range and intensive systems of poultry keeping should be educated by veterinary extension officers on the various kinds of gastrointestinal parasites in association with chickens and poultry as a whole and the dangers they pose.

The prevalence level shown by the birds in the study area is a clue to the susceptibility of the domestic fowl to many infectious diseases that may be detrimental to human consumption. It is therefore recommended that handlers and managers of poultry farms should improve upon their management skills and issues concerning hygiene. Veterinary Extension Officers are also requested to pay particular attention to the managerial practices of farmers in the area and provide the necessary assistance in protecting the health and wellbeing of poultry as well as contributing to public health protection. It is also recommended that further studies should be carried on the subject matter, covering different methods and parameters to enhance better results.

## Figures and Tables

**Table 1:** Prevalence of gastro-intestinal helminths of local and exotic breeds of chicken in Pankrono- Kumasi Ghana.

Parasite	No. Infected (n=131)	Prevalence in population (%)
*Ascaridia galli*	65	32.5
*Heterakis gallinarum*	38	19.0
*Capillaria *spp.	29	14.5
*Raillietina *spp.	19	9.5
*Choanotaenia infundibulum*	5	2.5
*Tetrameres americana*	5	2.5
*Davainea proglottina*	4	2.0
*Gongylonema ingluvocola*	4	2.0
* Prosthogonimus *spp.	3	1.5

## Data Availability

The data used to support the findings of this study are included in the article; however, the raw data is also available upon request from the corresponding author.
